# Living Through Decline: Resident Perceptions of Change Within Cleveland’s Urban Neighborhoods

**DOI:** 10.1093/geroni/igaa057.1417

**Published:** 2020-12-16

**Authors:** Kaitlyn Langendoerfer

**Affiliations:** Case Western Reserve University, Honesdale, Pennsylvania, United States

## Abstract

Despite the vast amount of research focused on neighborhoods within the environmental gerontology, very little attention has been paid to learning how older residents make sense of and describe the changes that have occurred within their communities over the course of their lives. The purpose of this study was to provide a space for residents to tell their stories of what it was like to live through neighborhood decline within Cleveland. Older adults are an ideal group for examining perceptions of neighborhood decline as they have the perspective to address both the historical changes of their neighborhood and the biographical changes of their lives. This study utilized data from 4 years of ethnographic observations with over 30 older (age 60+), African-American adults who have aged in place within Cleveland since their childhood. Additionally, multiple in-depth life history interviews were conducted with 13 long-term residents. Data was analyzed using grounded theory techniques for emergent themes. While each resident had their own unique perspective of neighborhood change, common themes emerged related to 1) institutional decline, 2) changes in safety and crime and 3) changes to the people living in their communities. The findings suggest that resident perceptions can help us better understand how neighborhood dynamics work their way into the lives of long-term residents. The results are particularly important as they provide the foundation for exploring how residents responded to neighborhood decline and why residents remained within their neighborhoods when so many others left.

